# Necrology

**Published:** 1895-06

**Authors:** 


### NECROLOGY.

Dr. Samuel Heiser, a well-known and respected veterinary surgeon of Sioux City, Iowa, died on the 5th of May.

Dr. George B. Burchsted, of New York City, a graduate of the American Veterinary College, Class of 1891, succumbed recently to diabetes at St. Luke's Hospital of the above city.

